# Preparation and Performance of Environmentally Friendly Micro-Surfacing for Degradable Automobile Exhaust Gas

**DOI:** 10.3390/polym17060760

**Published:** 2025-03-13

**Authors:** Tengteng Guo, Yuanzhao Chen, Chenze Fang, Zhenxia Li, Da Li, Qingyun He, Haijun Chen

**Affiliations:** 1 School of Civil Engineering and transportation, North China University of Water Resources and Electric Power, Zhengzhou 450045, China; guotth@ncwu.edu.cn (T.G.); fangchenze@126.com (C.F.); zhenxiali2009@ncwu.edu.cn (Z.L.); chenhaijun@ncwu.edu.cn (H.C.); 2 Technology Innovation Center of Henan Transport Industry of Utilization of Solid Waste Resources in Traffic Engineering, North China University of Water Resources and Electric Power, Zhengzhou 450045, China; 3 Henan Province Engineering Technology Research Center of Environment Friendly and High-Performance Pavement Materials, Zhengzhou 450045, China; 4 North China University of Water Resources and Electric Power-Henan Provincial Institute of Transportation Planning and Design Co., Ltd., Green, Low Carbon, and High Performance Road Materials Research and Development Center, Zhengzhou 450045, China

**Keywords:** pavement materials, TiO_2_/g-C_3_N_4_ composite photocatalyst, degradation of exhaust gas, microscopic characterization, composite-modified emulsified asphalt, road performance

## Abstract

To address the issue of air pollution caused by automobile exhaust in China, a titanium dioxide/graphite carbon nitride (TiO_2_/g-C_3_N_4_) composite photocatalyst capable of degrading automobile exhaust was prepared in this study. It was used as an additive to modify styrene–-butadiene latex (SBR) emulsified asphalt. The basic properties of modified emulsified asphalt before and after aging were analyzed, and the dosage range of TiO_2_/g-C_3_N_4_ (TCN) was determined. The environmentally friendly micro-surfacing of degradable automobile exhaust was prepared. Based on 1 h and 6 d wet wheel wear test, rutting deformation test, surface structure depth test, and pendulum friction coefficient test, the road performance of TCN environmentally friendly micro-surfacing mixture with different contents was analyzed and evaluated, and the effect of environmentally friendly degradation of automobile exhaust was studied by a self-made degradation device. The results show that when the mass ratio of TiO_2_ and melamine was 1:4, the TCN composite photocatalyst had strong photocatalytic activity. The crystal structure of TiO_2_ and g-C_3_N_4_ was not damaged during the synthesis process. The g-C_3_N_4_ inhibited the agglomeration of TiO_2_. The introduction of N-Ti bond changed the electronic structure of TiO_2_, narrowed the band gap and broadened the visible light response range. When the TCN content was in the range of 1~7%, the softening point of SBR- modified emulsified asphalt increased with the increase in TCN content, the penetration decreased, the ductility decreased gradually, and the storage stability increased gradually. The penetration ratio and ductility ratio of the composite-modified emulsified asphalt after aging increased with the increase in TCN content, and the increment of the softening point decreased. This shows that the TCN content is beneficial to the high-temperature performance and anti-aging performance of SBR-modified emulsified asphalt, and has an adverse effect on low temperature performance and storage stability. The addition of TCN can improve the wear resistance and rutting resistance of the micro-surfacing mixture, and has no effect on the water damage resistance and skid resistance. The environment-friendly micro-surfacing asphalt mixture had a significant degradation effect on NO, CO, and HC. With the increase in TCN content, the degradation efficiency of the three gases was on the rise. When the content was 5%, the degradation rates of NO, CO, and HC were 37.16%, 25.72%, and 20.44%, respectively, which are 2.34 times, 2.47, times and 2.30 times that of the 1% content, and the degradation effect was significantly improved.

## 1. Introduction

With the rapid development of the automobile industry, the number of automobiles in China is increasing, and it has gradually entered every family. Automobile emissions not only cause serious damage to human health by releasing harmful substances such as particulate matter (PM2.5) and nitrogen oxides (NOx) [1], but greenhouse gases such as CO_2_ and N_2_O, produced by them, also exacerbate global warming [2]. Automobile exhaust is an important cause of haze and photochemical smog. There are hundreds of polluting compounds in automobile exhaust, including CO, NOx, HC, PM2.5, and other polluting gases. From the analysis of the severity of human injury, CO, NOx, and HC are more serious, and may cause poisoning and even cancer [3]. As the carrier of motor vehicles, roads have the most direct contact with the exhaust gas of automobiles. The structure should have the use value in the direction of preventing and controlling the exhaust gas of automobiles. Therefore, the development of asphalt pavement that can degrade the exhaust gas of automobiles has unlimited environmental and social benefits.

TiO_2_ has a high electron yield, strong redox ability, and excellent chemical stability in nature, and is widely used in the field of photocatalytic air purification [4,5]. Carbon nitride is one of the oldest polymers. Compared with other photocatalytic materials, g-C_3_N_4_ has a narrow band gap, a wide range of visible light response, non-toxic and good biocompatibility, cheap raw materials, easy preparation, and good thermal and chemical stability [6,7]. Based on the respective properties of the two, the composite is modified to allow the composite material give full play to the synergistic effect between the two, and improve the photocatalytic activity. Ji et al. [8] used the precursor tetrabutyl titanate and urea to mix the two in advance, and then prepared g-C_3_N_4_/TiO_2_ by the solvothermal method. The results showed that the electron–hole pair recombination rate was reduced and the photocatalytic activity was improved. Zhao and Yan [9,10] chose titanium tetrachloride and melamine as raw materials to synthesize TiO_2_ and g-C_3_N_4_, respectively. Then, the two materials were mixed and calcined into TiO_2_/g-C_3_N_4_ composites. The decomposition of water to produce hydrogen and oxygen under natural light was studied. The results showed that the decomposition effect was significantly better than that of the two monomer materials. Guo Shuhui [11], Cang Jinshun [12], Li Junye [13], and Li Shufan [14] used industrial metatitanic acid, nano-TiO_2_, urea and melamine as raw materials. TiO_2_/g-C_3_N_4_, with better photocatalytic activity than the two monomer materials, was prepared by direct mixing and high-temperature calcination, and microscopic characterization was carried out. The experimental results were consistent, which proved that the performance of the composite material was the best.

Tan Yiqiu [15] used asphalt pavement as a carrier to study TiO_2_ in two ways: coating and incorporation. The results show that the degradation rate of NO can reach more than 80% under ultraviolet light. Hu et al. [16] added commercial anatase and rutile titanium dioxide in different proportions to waterborne epoxy resin, combined with asphalt pavement fog seal technology, and tested with a self-developed automobile exhaust decomposition device. The test results show that the NO, HC, and CO have a good decomposition effect, and can guarantee the anti-skid performance of the fog seal pavement and improve the anti-permeability of the pavement. Zhang [17] et al. designed a new photocatalytic micro-surfacing mixture (PMM) using polypropylene (PP) fiber and nano-titanium dioxide to enhance the photocatalytic effect. The results show that 0.2% PP fiber and 60% nano-titanium dioxide instead of mineral fillers is a feasible alternative method to improve the photocatalytic degradation of exhaust gas. Ma [18] et al. used nano-TiO_2_ modified asphalt in asphalt mixture, and studied and analyzed TiO_2_-modified pitch. It is evident from the findings that the incorporation of TiO_2_ notably enhances both the rheological characteristics and resistance to high-temperature rutting in asphalt. The asphalt mixture, having been modified with nano-TiO_2_, exhibits favorable properties for photocatalytically degrading both HC and NOx. Notably, the degradation efficacy observed in NOx was found to be significantly superior compared to that of HC. Yang [19] added nano-TiO_2_ loaded on tourmaline to SBR-emulsified asphalt for micro-surfacing. The augmentation of modified photocatalyst ameliorated the high temperature stability of SBR modified emulsified pitch, and the low temperature crack resistance was slightly reduced. It can still meet the demands of the specification, and its mixture has an excellent exhaust gas purification effect.

Ultimately, the synergistic combination of g-C_3_N_4_ with nano-TiO_2_ serves to address the individual limitations of each material, and gives full play to the synergistic effect between the two, so that it has better photocatalytic activity. The photocatalytic materials g-C_3_N_4_ and nano-TiO_2_ were separately applied to the degradation of automobile exhaust in pavement engineering. Compared with the modified photocatalytic materials, the degradation of tail gas was poor. At present, most of the research results are based on the analysis of exhaust gas degradation by coating. Research on the mixed sealing pavement is only limited to the addition of photocatalytic materials as mineral powder to the asphalt mixture. The application of the coating type in the pavement will be due to the wear of the wheels or the erosion of the rain. It is easy to lose and reduce its durability. In the application of the mixed type in the road engineering, because TiO_2_ is easy to gather and does not respond to visible light, it will cause the pavement to degrade the exhaust gas. There are few studies on adding photocatalytic materials as modifiers to emulsified asphalt, and then applying the modified emulsified asphalt to road engineering. Therefore, in this paper, the prepared photocatalytic material was used as a modifier in emulsified asphalt, and combined with micro-surfacing to prepare an environmentally friendly micro-surfacing that can degrade automobile exhaust so that the photocatalytic material can play a more effective degradation effect, and its road performance and degradation exhaust performance were studied.

## 2. Raw Materials

### 2.1. Photocatalytic Raw Material

In this paper, the anatase TiO_2_ produced by Jiangsu Tianxing New Materials Co., Ltd., Suzhou, China. was selected. The major technical indices are shown in Table 1. Melamine with a content of 99.5% was provided by Tianjin Damao Chemical Reagent Factory.

### 2.2. SBR-Modified Emulsified Asphalt

This study utilized styrene–butadiene rubber (SBR)-modified emulsified asphalt, a material supplied by Shanghai Yanzhixuan Nano New Materials Corporation, as a key component in the investigation. The SBR-modified emulsified asphalt was used to conduct routine tests on its basic performances according to the requirements of ‘Highway Engineering Asphalt and Asphalt Mixture Test Procedures’ (JTG E20-2011) [20], and its technical indicators were evaluated in accordance with the requirements of ‘Technical Guide for Micro-surfacing and Slurry Seal’ (2006) [21]. The outcomes of the tests are presented in Table 2 for reference. The observations indicate that the SB2R-modified emulsified asphalt satisfies the criteria outlined in the specifications [21].

### 2.3. Aggregate

In this paper, high-strength basalt was selected as a coarse aggregate, clean limestone as a fine aggregate, limestone powder as a mineral powder, so that the aggregate and emulsified asphalt have good adhesion and excellent abrasion resistance. According to the ‘Highway Engineering Aggregate Test Procedure’ (JTG E42-2005) [22], the test results and standard requirements are shown in Table 3, which meet the requirements of the micro-surfacing guide [21].

### 2.4. Cement

For this research, ordinary Portland cement of type P.O 42.5 was chosen as the primary binding material, and its main technical indicators are tested on the basis of the ‘Highway Engineering Cement and Cement Concrete Test Procedures’ (JTG E30-2005) [23]. Table 4 presents the outcomes of the performance evaluations conducted, and the technical performance indicators are qualified.

### 2.5. Water

The water for micro-surfacing mixture generally uses drinking water without harmful components, other pollutants, and chemical reaction substances. This article used Zhengzhou City’s drinking water, which meets the requirements of the specification [21].

## 3. Test Method

### 3.1. Test Method for the TiO_2_/g-C_3_N_4_ Composite Photocatalyst

#### 3.1.1. Preparation Method for the TiO_2_/g-C_3_N_4_ Composite Photocatalyst

**Figure 1:**
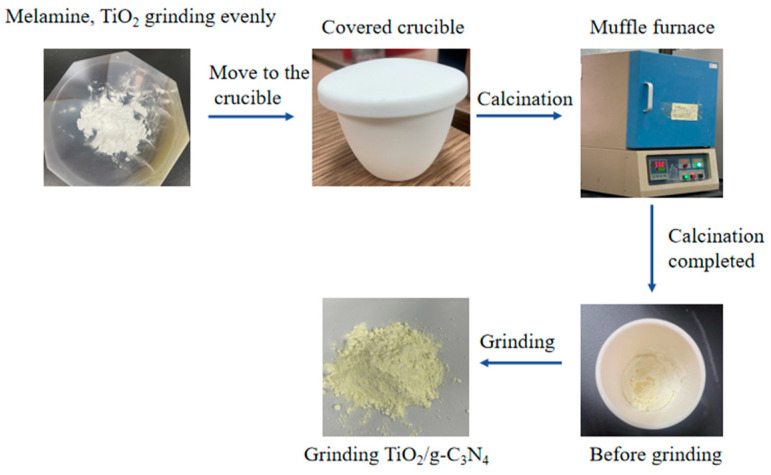
Preparation process of photocatalytic materials.

The TiO_2_/g-C_3_N_4_ composite photocatalyst was prepared using the high-temperature calcination method. Melamine and commercially available TiO_2_ were mixed according to a certain mass ratio and poured into the agate grinding bowl, then fully ground, transferred to a covered firepot, and shifted to a muffle kiln. The heating rate was set to 8 °C/min, the temperature was 550 °C, and the time was 4 h. After the high-temperature calcination and cooling, it was moved to the agate mortar to fully grind to obtain different TiO_2_/g-C_3_N_4_ composite materials. The schematic diagram is shown in Figure 1. The samples were labeled as TCN1, TCN2, TCN3, TCN4, and TCN5, according to the mass ratio, and TiO_2_ was used as the blank control group. The sample with the best photocatalytic activity was recorded as TCN for later analysis. Melamine was calcined under the same experimental conditions to generate graphite-phase carbon nitride (g-C_3_N_4_) for microscopic characterization.

#### 3.1.2. Analysis Method for the Photocatalytic Degradation Effect

**Figure 2:**
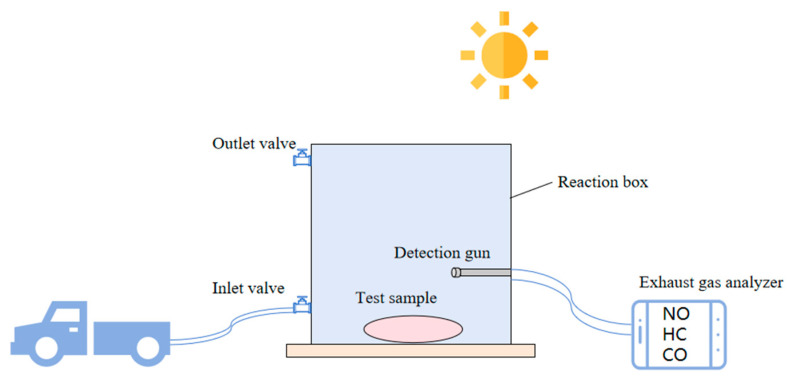
Photocatalytic degradation tail gas device.

With the purpose of evaluating and analyzing the degradation effect of photocatalytic materials on NO, CO, and HC in motor vehicle exhaust gas, a set of photocatalytic degradation devices was designed and assembled by our research group, including a gas reaction chamber, gas source channel, and real-time monitoring gas concentration equipment. The schematic diagram is shown in Figure 2. The experiments were conducted outdoors under natural sunlight between 10:30 and 15:00 Beijing time. In order to be closer to the composition of automobile exhaust, the gas source device selected 92# gasoline as the fuel of the Great Wall pickup truck. The lower side of the gas reaction chamber was an open square body with a size of l × b × h = 800 mm × 800 mm × 800 mm. The four sides and the top surface were made of organic glass. The thickness of the glass was 10 mm. It could observe to transmit over 92% of incident sunlight and 73.5% of ultraviolet radiation. The side was equipped with an intake valve, an exhaust valve, and a small hole. The bottom plate was a wooden board, and the size was larger than the bottom area of the reaction chamber. The polyurethane foam sealant was used to seal the test. The real-time gas concentration detection device selected the MKA-502 automobile exhaust gas tester of Foshan Hanchuang Detection Instrument Co., Ltd.

The specific test process of the photocatalytic degradation device designed in this paper is as follows:

(1) Check the airtightness of the reaction vessel to ensure that the reaction chamber is sufficiently sealed. Add 8 g of photocatalytic material to anhydrous ethanol, and put it into four petri dishes with a diameter of 180 mm on average. After ultrasonication for 10 min, put it in an oven at 80 °C for drying. Put the above-mentioned culture dish with the catalyst in the gas reaction chamber. If the test sample is a specimen, put it directly on the bottom plate, seal the reaction chamber, and seal it again with foam sealant. Initiate the procedure by opening both the inlet and outlet control valves. Subsequently, establish a connection between the air duct and the vehicle’s exhaust system. Finally, commence the automobile’s pre-heating phase, allowing it to run for a duration of 3 to 5 min.

(2) The detection gun of the exhaust gas analyzer is connected to the reaction chamber. At this time, the exhaust gas analyzer is opened for preheating and debugging, and then the reaction chamber is covered with a shading cloth. Through a large number of experiments in the early stage, it was found that the vehicle idle speed was maintained at 1200 r/min for about 20 min, and the gases in the reaction chamber were basically stable. The concentration range of each gas was shown in Table 5. At this time, the gases in the reaction chamber have reached a filling state, and the vehicle and the inlet and outlet control valves are closed.

(3) Allow for a 10 min interval to ensure uniform gas distribution within the reaction chamber. Following this, proceed to remove the light-blocking cover. Collect and document the initial concentrations of all gases present. Subsequently, terminate the instrumentation’s testing mode. The experiment was configured with a 90 min reaction duration, with data sampled at intervals of every 5 min.

(4) After the test, the exhaust valve was opened, and the automobile exhaust in the reaction chamber was emptied. The photocatalytic material samples or specimens are taken out to prepare for the next test.

(5) The cumulative degradation rate index was used as the evaluation standard, and the calculation formula was as follows (1):

(1) $$\eta =\frac{C_{0}-C_{t}}{C_{0}}$$

In the equation

*η*—Cumulative degradation rate (%);

*C*_0_—The initial concentration of exhaust gas (ppm/%);

*C*_t_—Tail gas concentration at time t (ppm/%).

#### 3.1.3. Microscopic Characterization of the TiO_2_/g-C_3_N_4_ Composite Photocatalyst

The X-ray diffraction (XRD) test, infrared spectroscopy (FTIR) test, scanning electron microscopy (SEM) test, transmission electron microscopy (TEM) test, ultraviolet-visible diffuse reflectance spectroscopy (UV-Vis DRS) test, and photoluminescence spectroscopy (PL) test were used to analyze the crystal structure, functional groups, morphological characteristics, the spectrum of light absorption, and the recombination velocity of photoexcited electrons and holes of the prepared photocatalytic materials.

### 3.2. Preparation of and Test Method for the SBR/TCN-Composite-Modified Emulsified Asphalt

#### 3.2.1. Preparation Method for the SBR/TCN-Composite-Modified Emulsified Asphalt

On the basis of the relevant literature [19], the content of TCN in this study was designed to be 1%, 3%, 5%, 7%, and 9% (percentage of emulsified pitch). The composite-modified emulsified asphalt was fabricated employing the technique of high-speed shearing. The emulsified bitumen was poured into a stainless steel vessel and heated to 80 °C. The constant temperature control heating platform was not started to prevent water evaporation during shearing. The 3% SBR latex and different amounts of TCN photocatalytic materials were added at a slow and uniform speed of 500 r/min. For a duration of 20 min, the shear rate was escalated to reach 5000 revolutions per minute, and the glass rod was continuously stirred during the period. After the shear, the modified emulsified asphalt was reduced to room temperature and placed in a container sealed for storage.

#### 3.2.2. Conventional Tests

According to the specification [20], the short-term aging test of SBR-modified emulsified asphalt (TCN photocatalytic material content is 0%) and (1%, 3%, 5%, 7%, 9%) TCN/SBR-composite-modified emulsified pitch was carried out by using an 85-type rotating film oven. Three major index tests were carried out on six groups of different modified pitch emulsion evaporation residues after aging, and the short-term anti-aging ability of each modified pitch emulsion was compared and evaluated. Combined with the storage stability test of modified emulsified asphalt before aging, the content range of the TCN material was determined.

### 3.3. Environmentally Friendly Micro-Surfacing Road Performance and Exhaust Gas Degradation Effect Research Methods

#### 3.3.1. Determination of Gradation

**Figure 3:**
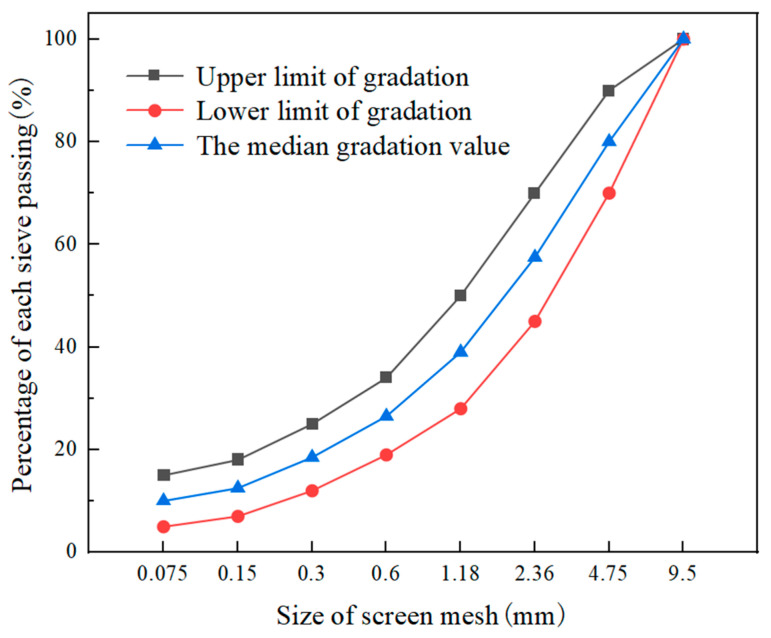
MS-3 micro-surfacing mineral aggregate gradation curve.

This study adopted the midpoint value within the gradation range specified for MS-3-type mineral aggregates in the respective standard as the basis for determining the gradation composition in designing the mixture proportions. Depicted in Figure 3 are the upper and lower boundaries defining the aggregate gradation, along with the composite gradation curve.

#### 3.3.2. Determination Method for Water Consumption

According to the specification [20], the water consumption of mixture was determined using the mixing test, and the micro-surfacing mixture with 3 different kinds of TCN was studied and analyzed. The oil–stone ratio of the mixing test was initially set to 7%, the cement dosage was 1.5%, and four different water consumptions of 5%, 6%, 7%, and 8% were taken to study and evaluate the influence of different water consumptions on the mixing time.

#### 3.3.3. Method for Determining the Amount of Cement

According to the specification [20], the amount of cement was determined by the cohesion test. The initial oil–stone ratio was 7%, the water consumption without photocatalytic material was 6%, and the water consumption with photocatalytic material was 7%. Different cement dosages (1%, 1.5%, 2%, 2.5%) were changed to study and analyze the influence of different cement dosages on the cohesion of environmentally friendly micro-surfacing mixture for 30 min and 60 min.

#### 3.3.4. Determination Method for the Optimum Oil–Stone Ratio

Conforming to the guidelines outlined in reference [20], this study employed the 1 h wet grinding wheel wear examination coupled with the load wheel sand testing diagram approach to ascertain the optimal oil-to-stone ratio range. The wet wheel wear experiment was to test the loss of automobile tires and pavement in the wet state, which was used to ascertain the minimum inclusion of modified emulsified pitch in micro-surfacing mixture, and evaluate the anti-wear and anti-water damage ability of micro-surfacing mixture and the consistency of mineral aggregate and emulsified pitch. The maximum inclusion of the modified emulsified pitch was ascertained by the load wheel sand experiment to prevent the occurrence of pavement oil spill caused by an excessive dosage. In this study, the water consumption of SBR-modified emulsified bitumen micro-surfacing mixture was 6%, the water consumption of micro-surfacing mixture with photocatalytic material was 7%, the cement content was 2%, and the different oil–stone ratios (6%, 6.5%, 7%, 7.5%, 8%) were changed. The 1 h wet wheel wear experiment and the load wheel sticky sand experiment were carried out, respectively.

#### 3.3.5. Road Performance Test Method

The 1 h, 6 d wet wheel wear experiment, rutting deformation experiment in specification [20], the pavement structure depth test in the ‘Highway Subgrade and Pavement Field Experiment Procedures’ (JTG 3450-2019) [24], the pendulum friction coefficient measurement method, the surface structure depth test, and the pendulum measurement experiment test were used to analyze and evaluate the wear counteract, water damage resistance, rutting deformation resistance, and anti-sliding performance of the TCN- and SBR-composite-modified emulsified bitumen micro-surfacing mixture with different dosages.

### 3.4. Tail Gas Degradation Effect Test

According to the self-made degradation device, degradation process and evaluation index in Section 3.1.2, the degradation exhaust effect of TCN composite photocatalytic material micro-surfacing mixture with different dosages of 1%, 3%, 5%, and 7% was analyzed. The micro-surfacing mixture was paved on the AC-13 waste rutting plate, about 10 mm thick, placed at indoor temperature (about 25 °C) for 24 h or more. Because of the large volume of the reaction box, it was necessary to place four rutting plates with environmentally friendly micro-surfacing mixtures.

## 4. Test Results and Analysis

### 4.1. Performance Analysis of the TiO_2_/g-C_3_N_4_ Composite Photocatalyst

#### 4.1.1. Effect of the Mass Ratio on Photocatalytic Activity

**Figure 4:**
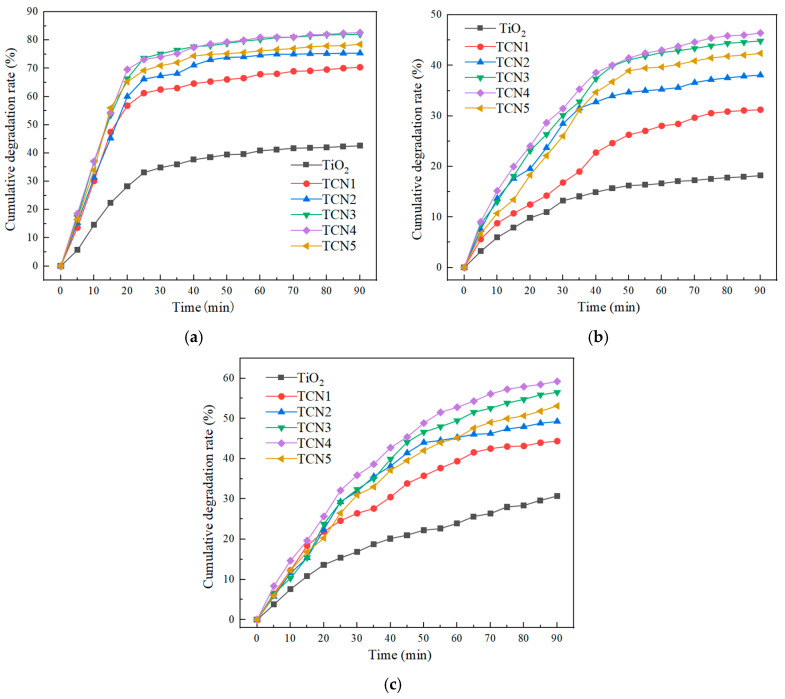
The cumulative degradation rate of NO, CO, and HC under different ratios. (**a**) Cumulative degradation rate of NO. (**b**) Cumulative degradation rate of CO. (**c**) Cumulative degradation rate of HC.

The cumulative degradation rates of three gases by the TiO_2_/g-C_3_N_4_ composite photocatalyst and anatase TiO_2_ prepared under different mass ratios under natural light are shown in Figure 4a–c.

As shown in Figure 4a–c, the TiO_2_/g-C_3_N_4_ composite photocatalytic material exhibited an excellent decomposition effect on NO, CO, and HC, although the degradation efficiency varies significantly among the three gases. The degradation effect of NO was decidedly better than that of CO and HC. From the degradation of NO, CO, and HC, when the mass ratio of melamine to TiO_2_ was 4:1, the degradation efficiency was the highest, which was 82.68%, 59.25%, and 46.42%, respectively. Its photocatalytic property is far more than that of the TiO_2_ monomer material, and the degradation rate was 1.94 times, 1.93 times, and 2.55 times of TiO_2_, respectively. It can be seen that the TiO_2_/g-C_3_N_4_ composite photocatalytic material did not simply superimpose the two materials, but played a synergistic role between TiO_2_/g-C_3_N_4_ and significantly improved its photocatalytic activity. When the mass ratio of the two was too high, a small amount of TiO_2_ made the photogenerated electrons formed by g-C_3_N_4_ unable to efficiently transition to the surface of TiO_2_, which decreased the separation rate of photogenerated hole–electron pairs, thus affecting the photocatalytic activity of the composites. When the mass ratio of the two was too low, the gas degradation efficiency was reduced compared with TCN4 because the content of g-C_3_N_4_ in the TiO_2_/g-C_3_N_4_ material was lower than that of TCN4.

#### 4.1.2. Influence of Calcination Temperature on Photocatalytic Activity

**Figure 5:**
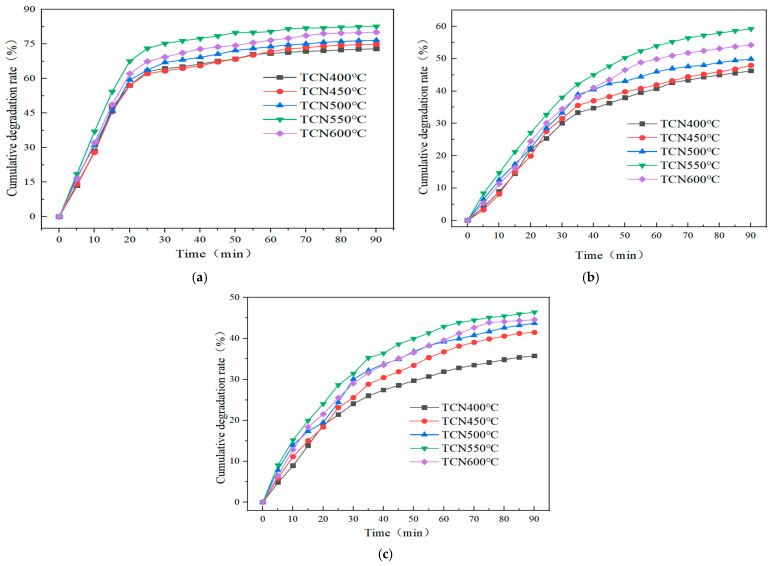
The cumulative degradation rate of NO, CO, and HC at different temperatures. (**a**) Cumulative degradation rate of NO. (**b**) Cumulative degradation rate of CO. (**c**) Cumulative degradation rate of HC.

The cumulative degradation rates of three gases (NO, CO, and HC) by the prepared TiO_2_/g-C_3_N_4_ composite photocatalysts calcined at different temperatures under natural light are shown in Figure 2, Figure 3, Figure 4 and Figure 5a–c.

It can be concluded from Figure 5a–c that the cumulative degradation rates of the three gases increased with the rise in temperature. The degradation efficiencies in descending order according to temperature were 550 °C > 600 °C > 500 °C > 450 °C > 400 °C. The cumulative degradation rate reaches the maximum at 550 °C. This is because as the temperature increases, both the pore size and specific surface area of the TiO_2_/g-C_3_N_4_ material increase, providing more active adsorption sites and centers, and thus enhancing its photocatalytic activity. The degradation efficiency at 400 °C was the lowest, with the cumulative degradation rates of NO, CO, and HC being 72.98%, 46.25%, and 35.71%, respectively. This is due to the insufficient calcination of melamine at such a low temperature, resulting in the residue of impurities that cover the surface of the composite material, thereby affecting the photocatalytic activity. The degradation efficiency at 600 °C is lower than that at 550 °C. This is because an excessively high temperature can cause the decomposition of the generated g-C_3_N_4_, leading to a relatively low content of g-C_3_N_4_, and thus affecting the photocatalytic activity of the composite photocatalyst.

#### 4.1.3. X-Ray Diffraction (XRD) Analysis

**Figure 6:**
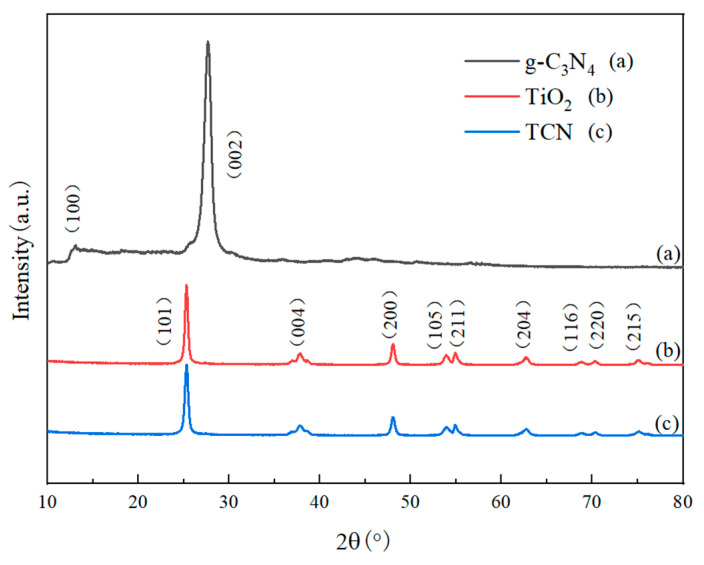
XRD patterns of TiO_2_, g-C_3_N_4_, and TCN.

From Figure 6 showing the XRD charts of pure TiO_2_, pure g-C3N4, and TCN, it can be seen that TiO_2_ was a typical anatase phase, and there were sharp diffraction maximums at 2θ = 25.36°, 37.82°, 48.20°, 53.87°, 54.91°, 62.70°, 68.84°, 70.31°, and 75.15°, which corresponded to (101), (004), (200), (105), (211), (204), (116), (220), and (215) lattice planes of anatase TiO_2_, severally. There were no characteristic peaks of other crystal forms, indicating that it had good crystallinity. The characteristic peaks of g-C_3_N_4_ appeared at 13.11° and 27.39°. The characteristic peak of 13.11° was formed by the accumulation of an in-plane tri-s-triazine ring motif structure, which corresponded to the (100) crystal plane of g-C_3_N_4_. The characteristic peak of 27.39° was present because of the accumulation of aromatic compounds in the ringed adjoint system between the characteristic layers of g-C_3_N_4_, relating to the (002) crystal plane of g-C_3_N_4_. From the XRD diffraction pattern of the TCN samples, it can be found that the diffraction peak position was basically unchanged compared with pure TiO_2_, indicating that the addition of g-C_3_N_4_ had little effect on the crystal structure of TiO_2_. The characteristic peaks of 13.11° and 27.39° of g-C_3_N_4_ did not appear in the composites. There are two factors: one is that nano-TiO_2_ was attached to the surface of g-C_3_N_4_, so that the diffraction peak of g-C_3_N_4_ was weakened and it was difficult to be detected. The other is that the crystal structure of nano-TiO_2_ was less affected during the high-temperature calcination process, resulting in the characteristic peak intensity of g-C_3_N_4_ being obscured by the characteristic peak intensity of TiO_2_.

#### 4.1.4. Infrared Spectroscopy (FTIR) Analysis

**Figure 7:**
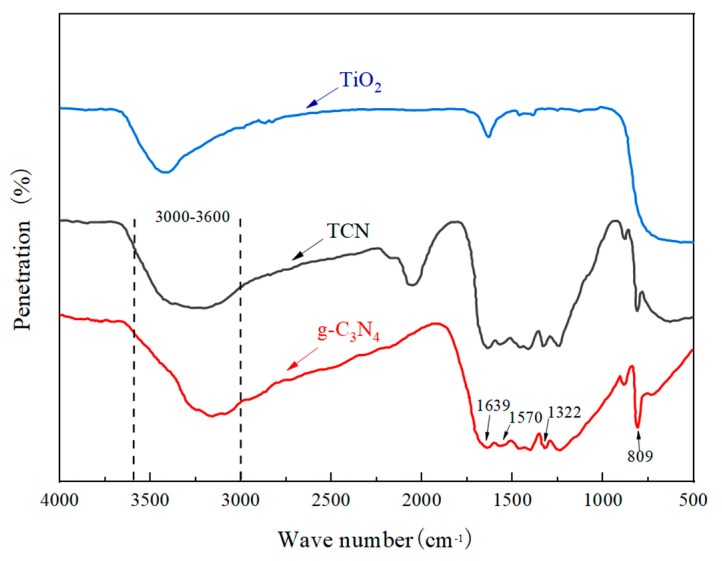
FTIR spectra of TiO_2_, g-C_3_N_4_, and TCN.

Figure 7 is the FTIR diagram of pure TiO_2_, pure g-C_3_N_4_, and TCN. It can be seen from Figure 7 that pure g-C_3_N_4_ had characteristic peaks at 809 cm^−1^, 1200 cm^−1^~1700 cm^−1^, and 3000 cm^−1^~3600 cm^−1^. This obvious trough at 809 cm^−1^ is attributed to the bending vibration of C-N or C=N in the triazine ring structure, which further confirms that the product obtained by calcination was a graphite-like carbon nitride structure; multiple fluctuation peaks appeared in the wavelength range of 1200 cm^−1^~1700 cm^−1^, which were caused by the tensile vibration of the typical heptazine ring unit (C6N7). The characteristic peaks of 1238 cm^−1^ and 1322 cm^−1^ were caused by the stretching vibration of C-NH-C or C-N-C unit. The characteristic peaks of 1405 cm^−1^, 1463 cm^−1^, 1570 cm^−1^, and 1639 cm^−1^ are attributed to the stretching vibration of C-N bond and C=N bond on the carbon–nitrogen heterocyclic ring. The characteristic peaks in the range of 3000 cm^−1^~3600 cm^−1^ are attributed to the stretching of O-H or N-H of the NH_2_ or NH group connected to the C atom, which was formed by the hybridization of atomic orbitals. Nano-TiO_2_ had a wider absorption peak in the range of about 525 cm^−1^, which was due to the stretching vibration of Ti-O and Ti-O-Ti units. The characteristic peaks at 1628 cm^−1^ and 3426 cm^−1^ were due to a small amount of adsorbed water or O-H stretching vibration. The weak absorption band of TCN at a wavenumber of 2100 cm^−1^ is attributed to the C≡N stretching vibration of the un-condensed melamine precursors or intermediates remaining during the synthesis of g-C_3_N_4_. This peak was absent in pure TiO_2_ and not very obvious in pure g-C_3_N_4_ (Figure 6), indicating that the calcination process partially retained the cyanide groups at the TiO_2_/g-C_3_N_4_ interface. The incomplete polycondensation of melamine resulted in a small amount of C≡N residues, which did not affect the photocatalytic performance of the composite material, as evidenced by its superior degradation efficiency compared to the individual components (Section 4.1.1). The leading feature peaks of TiO_2_ and g-C_3_N_4_ appeared in the FTIR spectrum of TCN, and no other characteristic peaks appeared, showing that the two materials of TiO_2_ and g-C_3_N_4_ were successfully compounded, and the structure was not damaged during the composite calcination process.

#### 4.1.5. Scanning Electron Microscopy (SEM) Analysis

**Figure 8:**
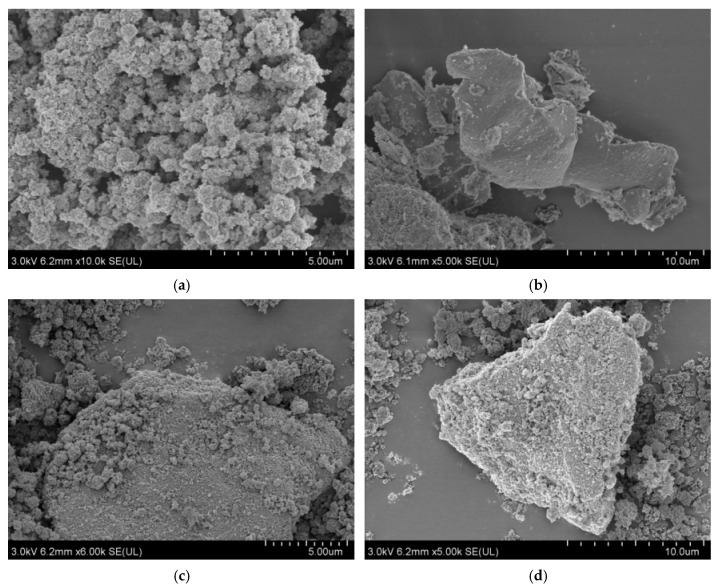
SEM images of the three photocatalytic materials: (**a**) TiO_2_; (**b**) g-C_3_N_4_; (**c**) TCN; (**d**) TCN.

Figure 8a shows the SEM picture of TiO_2_. It can be drawn that TiO_2_ particles are approximately spherically stacked together, and the agglomeration phenomenon is more serious. This is attributed to the small diameter of TiO_2_ nanoparticles, the surface energy being large, and it being easy to agglomerate, which affects the photocatalytic activity of TiO_2_. From the SEM image of g-C_3_N_4_ in Figure 8b, it can be seen that the material was lamellar and the surface was relatively smooth and flat, indicating that melamine had generated graphite-like lamellar g-C_3_N_4_ by high-temperature thermal polymerization. Figure 8c,d shows the SEM picture of TCN composite photocatalytic materials under the optimal preparation process. It can be seen that TiO_2_ particles were uniformly distributed on the surface of the g-C_3_N_4_ sheet structure, and the small particles show a highly dispersed state. There was no agglomeration phenomenon, and g-C_3_N_4_ was not completely covered, indicating that the presence of g-C_3_N_4_ inhibited the agglomeration of TiO_2_. Therefore, the composite photocatalytic material TCN had a big surface area, thereby increasing the photocatalytic activity of TiO_2_, the contact area with the polluted gas increased, and the degradation rate increased.

#### 4.1.6. Transmission Electron Microscope (TEM) Analysis

**Figure 9:**
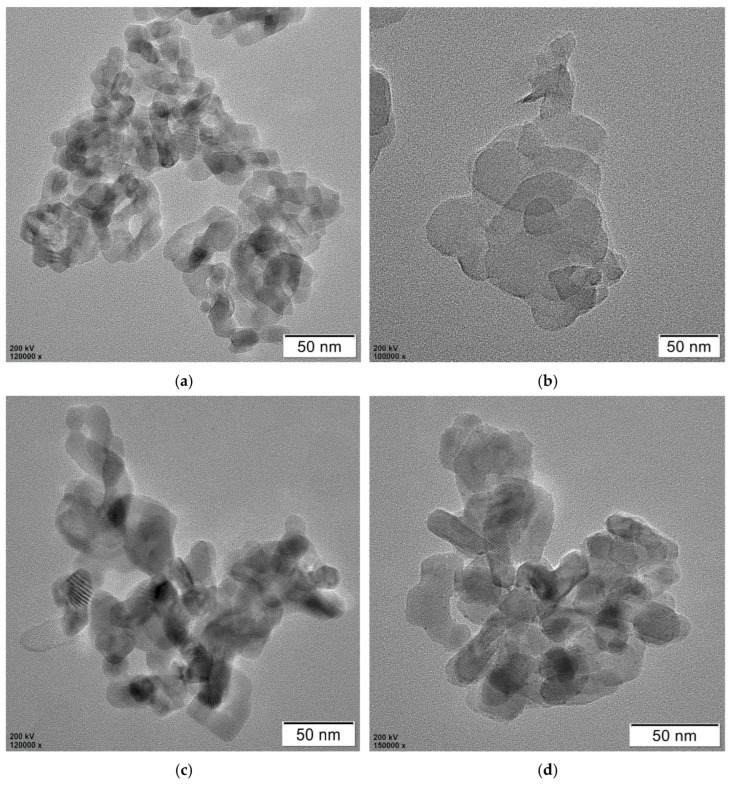
TEM images of the three photocatalytic materials: (**a**) TiO_2_; (**b**) g-C_3_N_4_; (**c**) TCN; (**d**) TCN.

The TEM images depicted in Figure 8 illustrate pure TiO_2_, pure g-C3N4, and TCN. It can be observed from Figure 9a that the TiO_2_ structure was stacked by a large number of small particles, and each particle was closely connected and the agglomeration phenomenon was more serious. From the transmission electron microscope image of g-C_3_N_4_, it can be seen that it presented a thin yarn sheet structure with a lighter color. From Figure 9c,d, it can be fairly seen that the distribution of nano-TiO_2_ particles on the layered structure of g-C_3_N_4_ sheet, which was evenly dispersed, reduced the agglomeration phenomenon and formed a closely connected composite with g-C_3_N_4_ sheet. The TEM image of the TCN composite photocatalytic material was consistent with the scanning electron microscope test image, which proves that the two materials were successfully compounded. From Figure 9c,d, it can also be seen that the g-C_3_N_4_ in TCN was not completely covered by TiO_2_. At the same time, due to the attachment of TiO_2_ particles, the surface of TCN formed voids of different sizes and shapes, which increased the surface area of the bonded material. It not only did not affect its absorbance, but also increased the contact area with automobile exhaust, and could capture photogenerated electrons, increase carrier separation rate, and improve photocatalytic activity.

#### 4.1.7. UV-Vis Diffuse Reflectance Spectroscopy (UV-Vis DRS) Analysis

**Figure 10:**
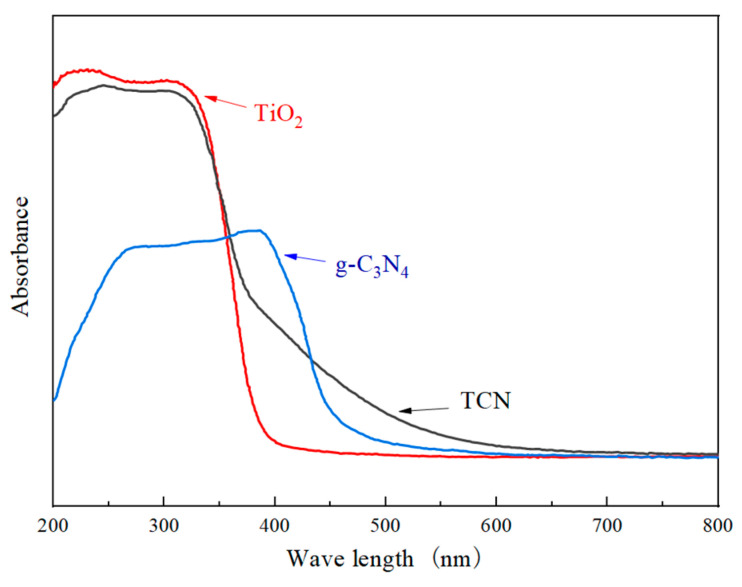
UV-visible diffuse reflectance spectra.

**Figure 11:**
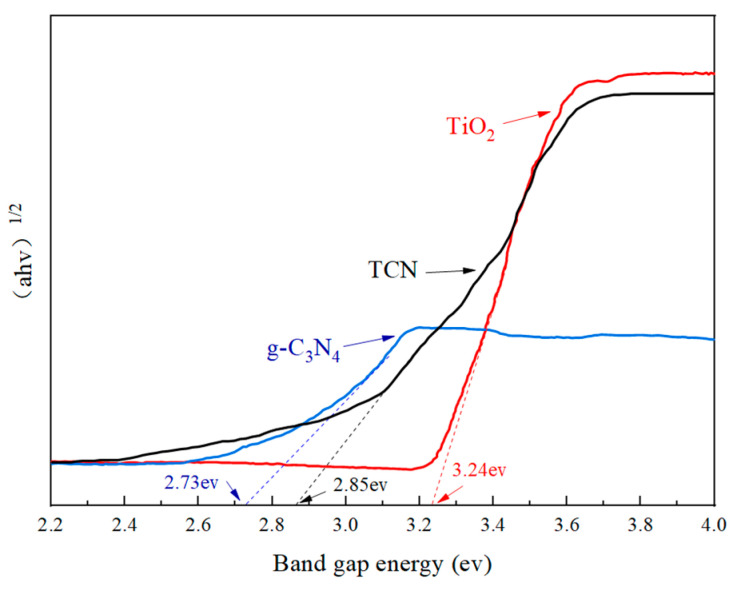
Band gap energy diagram.

Figure 10 illustrates that the pure TiO_2_ ceased to exhibit light absorption at a wavelength of approximately 383 nm, a value that aligns with the inherent absorption edge of anatase-phase TiO_2_. In the ultraviolet light range of 200 mm~380 nm, the absorbance of TiO_2_ was greater than that of TCN and g-C_3_N_4_, which is related to the characteristics of TiO_2_ itself. Within the visible light spectrum spanning 400 nm to 600 nm, the observed absorbance was diminished compared to both TCN and unadulterated g-C_3_N_4_. The light reaction range of the TCN composite material was 200 nm~600 nm, which is superimposed with the light response range of g-C_3_N_4_ and TiO_2_. Compared with g-C_3_N_4_ and TiO_2_, there is a significant red shift, which allows it to have excellent light response intensity. It can be seen from Figure 11 that the band gaps of TiO_2_, g-C_3_N_4_, and TCN were 3.24 eV, 2.73 eV, and 2.85 eV, respectively. The energy gap of the composite photocatalytic material TCN was between TiO_2_ and g-C_3_N_4_, and its value was 0.39 eV lower than that of pure TiO_2_. The incorporation of g-C_3_N_4_ suggests the formation of N-Ti bonds, leading to modifications in TiO_2_’s electronic structure. This alteration reduced the band gap energy, thereby enhancing the visible light absorbance capability of the composite material, TCN. Consequently, the photocatalytic redox potential of the material was significantly augmented.

#### 4.1.8. Photoluminescence Spectroscopy (PL) Analysis

**Figure 12:**
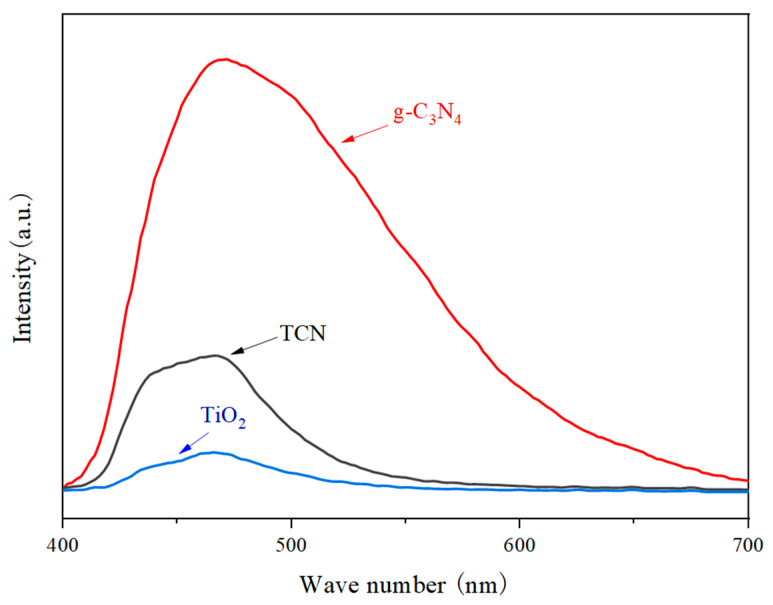
PL spectra of TiO_2_, g-C_3_N_4_, and TCN.

It can be concluded from the photoluminescence Figure 12 that the PL peak intensity was ranked as g-C_3_N_4_ > TCN > TiO_2_, and the corresponding electron–hole pair recombination rate was also ranked as g-C_3_N_4_ > TCN > TiO_2_. The reason for the low intensity of the fluorescence characteristic peak of TiO_2_ is that the test light was visible light and TiO_2_ did not respond to it. Notably, the fluorescence intensity peak of TCN was observed to be substantially diminished compared to that of g-C_3_N_4_, suggesting that the integration of TiO_2_ with g-C_3_N_4_ effectively facilitated the formation of a heterojunction interface. This interface, in turn, played a pivotal role in enhancing the separation efficiency of photoinduced electron–hole pairs, which was mainly due to the redox potential mutual action between the valence band of TiO_2_ and the conduction band of g-C_3_N_4_; that is, the photogenerated electrons were transferred from g-C_3_N_4_ to TiO_2_, while the holes were transferred from TiO_2_ to g-C_3_N_4_, which created favorable conditions for improving the photocatalytic performance of TiO_2_ and enhanced the photocatalytic degradation of pollutants by TiO_2_.

### 4.2. Performance Analysis of the SBR/TCN-Composite-Modified Emulsified Asphalt

#### 4.2.1. Basic Performance Analysis Before Aging

The three indicators of SBR/TCN-composite-modified asphalt emulsion evaporation residue and the influence of storage stability were studied and analyzed. According to the test procedure [20], the property test of composite modified asphalt emulsion was carried out. The findings are depicted in Table 6.

The gradual decrease in penetration observed with increasing doses of TCN suggests that the incorporation of photocatalytic materials has a viscosity-enhancing effect on the emulsified asphalt to some extent. Compared with SBR modified emulsified pitch, the penetration of 9%TCN + 3%SBR-composite-modified asphalt emulsion declined by 0.65 mm, and the decrease reached 10.1%. However, the decrease of 7~9% was small, which was 0.08 mm lower than that of 7%. Observably, the enhancement in emulsified asphalt performance demonstrates a tendency toward saturation as the content reaches excessively high levels. Notably, an increase in the TCN content corresponded to a rise in the softening point of the composite-modified emulsified asphalt, suggesting that the incorporation of TCN photocatalytic materials imparts a stiffening impact on the emulsified asphalt. This augmentation in stiffness leads to an elevation in the viscosity of the emulsified asphalt mortar, consequently enhancing the high-temperature performance characteristics of the modified emulsified asphalt. In the process of increasing the amount of TCN, the ductility decreased continuously, and the ductility decreased greatly. Compared with SBR-modified asphalt emulsion, it was reduced by 21 cm at most. This is because when the amount of photocatalytic material is too much, a part of it will be free in the emulsified asphalt, occupying a certain volume, so that the effective asphalt in the unit volume is reduced, the fracture speed is accelerated, and brittle fracture is easy to form, so that the low temperature cracking performance of emulsified asphalt is decreased, but all meet the construction requirements of more than 20 cm.

With the increase in photocatalytic materials, the storage performance of composite-modified emulsified pitch gradually deteriorated. When the mixing amount of photocatalytic material is 9%, the storage performance of composite-modified emulsified asphalt for 1 d and 5 d exceeds the standard requirements of 1% and 5%. This is due to the excessive amount of doping, which will make some photocatalytic materials unable to fully integrate with asphalt, resulting in the acceleration of emulsion sedimentation speed and poor stability. In summary, the addition of a TCN composite photocatalytic material can increase the high-temperature performance of composite-modified emulsified pitch, but it has an adverse effect on the low-temperature anti-cracking property, and the addition of an SBR latex can make up for this shortcoming. The increase in excessive TCN composite photocatalytic materials tended to be saturated with performance improvement and had an adverse effect on storage stability. Therefore, it is recommended to use 1~7% TCN to modify emulsified asphalt for the study of automobile exhaust degradation by an environmentally friendly micro-surfacing mixture.

#### 4.2.2. Basic Performance Analysis After Aging

To evaluate the short-term anti-aging performance of each modified emulsified asphalt, an analysis was conducted on three key parameters: the ratio of residual penetration, the increment in softening point, and the ratio of residual ductility. These parameters were examined for the evaporation residues of six distinct groups of modified emulsified asphalt following aging procedures. A comparative assessment is presented based on these findings, as illustrated in Table 7.

With the addition of nano-TCN composite photocatalytic materials, the penetration ratio and ductility ratio of composite modified emulsified asphalt increased after senescence, and the 7% TCN content before showed a rising trend. The penetration ratio increased from 69.10% to 74.79%, and the ductility ratio increased from 68.02% to 73.86%. When the content reached 9%, the ratio decreased, but the penetration ratio and ductility ratio of SBR-modified asphalt emulsion were still increased compared with SBR-modified asphalt emulsion, indicating that the addition of a nano-photocatalytic material TCN is good for the modified asphalt emulsion of SBR/TCN-composite-modified emulsified asphalt. The increase in the consistency of the evaporated residue after aging will increase the softening point. The softening point of six groups of emulsified bitumen increased, and the increment of the softening point decreased from 6.9 °C to 3.7 °C before the content of the nano-TCN composite photocatalytic material was 7%. When the content reached 9%, there was a reverse increase. Compared with SBR-modified asphalt emulsion, the softening point increment was still reduced, indicating that the addition of a TCN composite photocatalytic material can increase the aging-resistant performance of a composite-modified emulsified pitch.

In summary, the nano-photocatalytic material TCN can improve the anti-aging ability of the composite-modified emulsified bitumen to a certain degree. Similar to the conclusions drawn by relevant scholars [25,26], the main factor is that the TCN photocatalytic material itself has a unique interface effect, and the absorption and shielding functions of visible light and ultraviolet light, but when TCN reaches a certain amount, it will be unevenly dispersed and cause agglomeration, thereby reducing the space for effective asphalt and reducing its own anti-aging performance. Therefore, combined with the previous analysis of the basic performances of a composite-modified asphalt emulsion, this paper selected 3%-SBR- and 1~7%-TCN-photocatalyst-content-composite-modified emulsified asphalt for the following research.

### 4.3. Analysis of a Mix Proportion Design of Environmentally Friendly Micro-Surfacing

#### 4.3.1. Determination of Water Consumption

**Figure 13:**
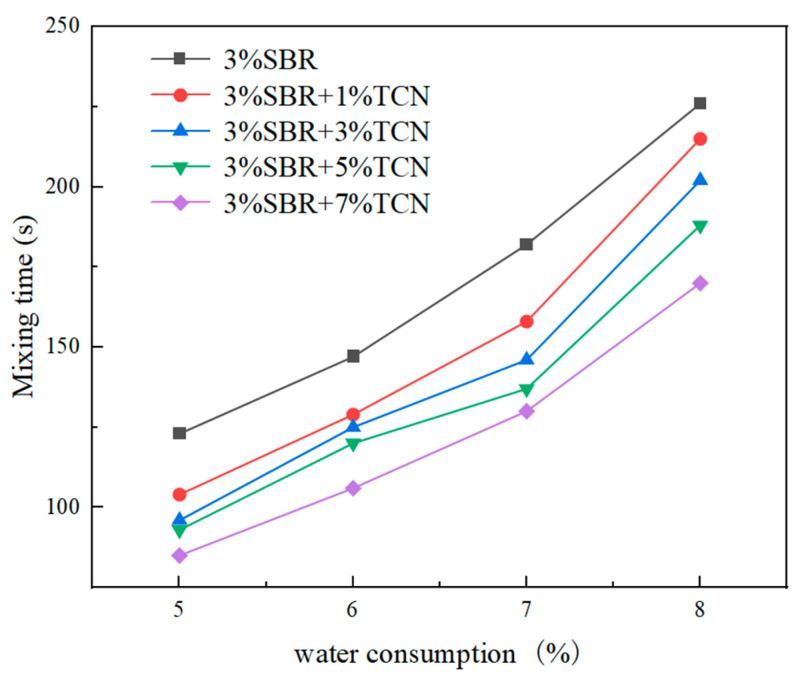
Mixing time test results of the mixture with different TCN material contents.

The asphalt–stone ratio was initially set as 7%, and the cement content was 1.5%. The mixing experiment was carried out on the micro-surfacing with different water consumptions of 5%, 6%, 7%, and 8%. The mixing time experiment results of the micro-surfacing mixture with different TCN material contents are shown in Figure 13.

It can be known from Figure 13 that with the added water consumption, the fixing time was gradually prolonged. This is mainly because the diffusion layer in the electric double layer of emulsified asphalt expanded due to the increase in water, and the corresponding potential increased. The modified emulsified asphalt was further diluted, so that the collision rate between the asphalt particles was reduced and the demulsification time was delayed. When the amount of added water was the same, the fixing time of the micro-surfacing mixture with photocatalytic materials was reduced, and there were some differences in the mixing time of different photocatalytic materials. When the water consumption was more than 7%, the SBR-modified emulsified asphalt mixture was too thin, resulting in segregation and flow phenomenon, and the mixing time could reach more than 180 s, which is much longer than 120 s; when the water consumption was 6%, the slurrying state of the mixture was good.

Adding different amounts of photocatalytic material micro-surfacing mixture, when the water content was less than 6%, the slurry state was relatively trunk, and the fixing time was less than 120 s, as required by the specification. When the water content reached 8%, the mixture appeared in a segregation state. This is because when the water added is too little, the aggregate surface is not fully soaked, and the aggregate will absorb the water in the emulsified bitumen, resulting in the early demulsification time of the emulsified bitumen; the mixture becomes thicker and is not easy to mix. The corresponding mixing time was shortened, and the slurry state was not good; when the water consumption is too large, excess water will be produced, which will adversely affect the mixture. It can be seen from this that when the additional water consumption is too low, the consistency of the mixture will increase, resulting in insufficient paving time and poor paving conditions, resulting in low adhesion to the road surface and making it easy to fall off. When the additional water consumption is too high, the consistency of the mixture will be reduced to make it too thin, and the segregation phenomenon of aggregate and asphalt layering will occur, which will affect the construction workability of the mixture and reduce the later strength. Therefore, it is necessary to select the appropriate additional water consumption to ensure that the micro-surfacing mixture has good construction performance and sufficient strength. In summary, the optimal water consumption of the micro-surfacing mixture without and with TCN photocatalytic materials was 6% and 7%, respectively.

#### 4.3.2. Determination of Cement Dosage

**Figure 14:**
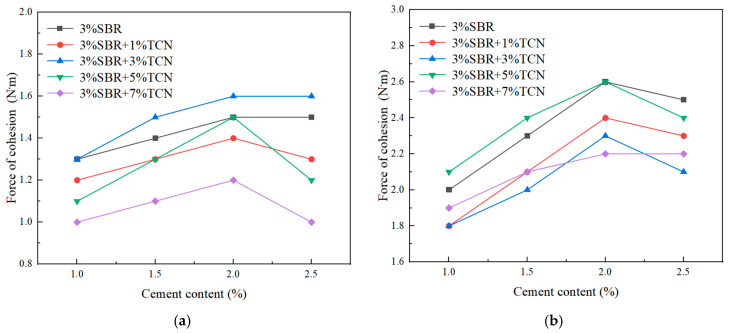
Cohesion test results of mixtures with different TCN material contents: (**a**) 30 min cohesion; (**b**) 60 min cohesion.

The asphalt–stone ratio was 7%, the water consumption without photocatalytic material was 6%, the water consumption with photocatalytic material was 7%, and the different cement dosages (1%, 1.5%, 2%, 2.5%) were changed. The micro-surfacing mixture with different cement composition and different TCN material contents was subjected to 30 min and 60 min cohesion tests. The outcomes of the examination are depicted in Figure 14.

From the analysis of Figure 14, it can be seen that when other conditions remained unchanged and the cement content was less than 2%, the cohesion of the micro-surfacing mixture at 30 min and 60 min increased with the increase in the cement content. When the cement content was greater than 2%, there was a decline or gentle phenomenon. The reason is that when the amount of added water is constant, the amount of cement added is too small, the water demand in the hydration reaction process is less, the water is not easy to evaporate, and the mixture will have excess free water. The aggregate was surrounded by water for a long time, which delayed the contact with emulsified asphalt, prolonged the demulsification time of emulsified asphalt, and thus reduced the early intensity of the mixture. With the improvement in cement constituents, the heat generated in the hydration process evaporated the free water in the mixture, promoted the early demulsification of emulsified bitumen, increased the force between aggregate and emulsified asphalt, and the interaction between the product of cement hydration and asphalt improved the early strength of the mixture and shortened the open traffic time. When the cement content exceeded a certain value, the amount of water in the mixture could not meet the water demand for cement hydration heat production, causing some cement to be insufficiently hydrated and agglomerated, resulting in defects in the formed mixture, weakening the early strength of the mixture, and even causing cracks. According to the results of the 30 min and 60 min cohesion tests, when the cement content was 2%, the mixture had good cohesion and met the standard requirements. Therefore, this paper chose 2% cement content.

#### 4.3.3. Determination of the Best Oil–Stone Ratio

**Figure 15:**
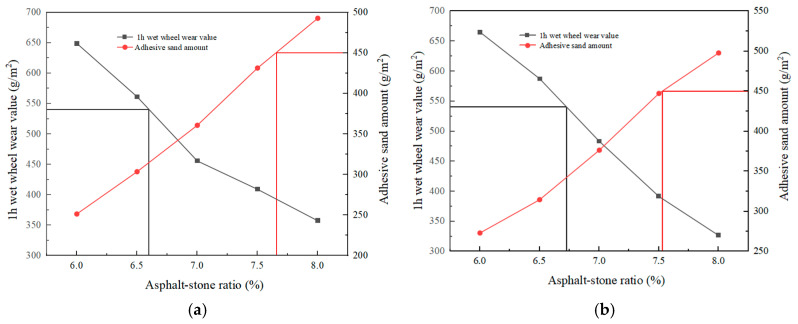

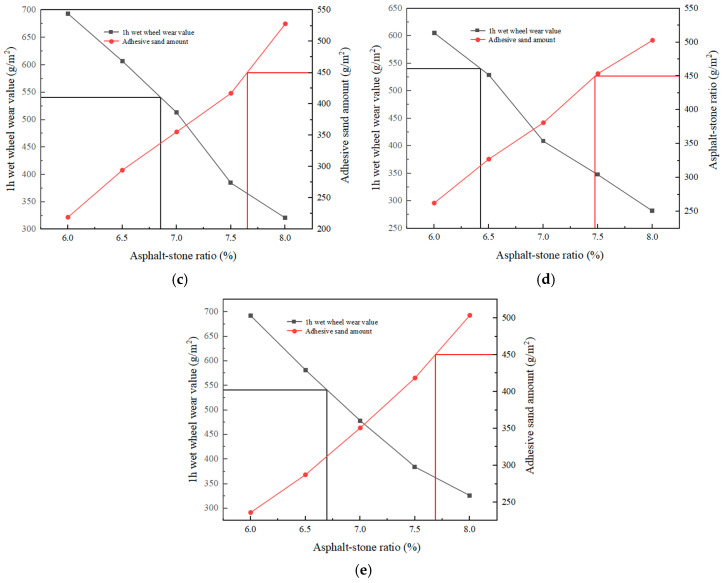
Oil–stone ratio curve of mixture with different TCN material contents: (**a**) 3%SBR-modified emulsified asphalt; (**b**) 3%SBR + 1%TCN-modified emulsified asphalt; (**c**) 3%SBR + 3%TCN-modified emulsified asphalt; (**d**) 3%SBR + 5%TCN-modified emulsified asphalt; (**e**) 3%SBR + 7%TCN-modified emulsified asphalt.

The water consumption of the SBR-modified emulsified bitumen micro-surfacing mixture was 6%, the water consumption of the micro-surfacing mixture with photocatalytic material was 7%, the cement dosage was 2%, and the different oil–stone ratios (6%, 6.5%, 7%, 7.5%, 8%) were changed. The 1 h wet wheel wear test and the load wheel sticky sand test were carried out, respectively. According to the specification, the test data were drawn into a diagram, and the most excellent oil–stone ratio range was obtained. The drawing is shown in Figure 15.

It can be known from Figure 15 that with the improvement in the oil–stone ratio, the 1 h wet wheel wear value and the load wheel sand content showed a downward and upward trend, respectively. From Figure 4, Figure 5, Figure 6, Figure 7 and Figure 8, the most excellent oil–stone ratio range of different micro-surfacing mixtures can be obtained. The most excellent oil–stone ratio range of micro-surfacing mixtures with different TCN contents was as follows: 3% SBR-modified emulsified asphalt 6.60~7.66%; 3%SBR + 1%TCN-modified emulsified asphalt 6.73~7.53%; 3%SBR + 3% TCN-modified emulsified asphalt 6.85~7.65%; 3% SBR + 5% TCN-modified emulsified asphalt 6.43~7.47%; and 3%SBR + 7% TCN-modified emulsified asphalt 6.70~7.69%. In order to facilitate the comparative analysis later, according to the specification requirements, the optimal oil–stone ratio of the five different micro-surfacing mixtures in the curve was 7.2%. The micro-surfacing mixture was mixed with this asphalt–stone ratio, and the subsequent performance test was carried out.

### 4.4. Environmentally Friendly Micro-Surfacing Road Performance Analysis

#### 4.4.1. Wear Resistance Performance Analysis

To assess the wear resistance of the micro-surface, a 1 h wet wheel wear test was employed. Here, a higher measured wear value indicates diminished wear resistance, whereas a lower value signifies enhanced resilience against wear. The outcomes of this testing are summarized in Table 8.

From the data presented in Table 8, it becomes evident that the 1 h wet wheel abrasion value of the specimens exhibited a gradual decline as the content of TCN composite photocatalytic material ranged from 0% to 5%. However, at a 7% content level, there was a conspicuous rise in the wear value, albeit it managed to satisfy the specified requirement of 540 g/m^2^. The 1 h wet wheel wear value of 1%, 3%, and 5% was reduced by 11.5 g/m^2^, 17.7 g/m^2^, and 26.4 g/m^2^, respectively, compared with 0%. When the content increased from 5% to 7%, the wet wheel wear value increased by 37.6 g/m^2^. It can be seen that the addition of an appropriate amount of photocatalytic materials does not weaken the wear performance of the micro-surface mixture. On the contrary, it had a slight improvement effect on the wear ability of the micro-surface. An excessive addition of photocatalytic materials had an adverse influence on the wear ability of micro-surfacing mixture, but it still met the specification requirements.

#### 4.4.2. Analysis of Water Damage Resistance

The evaluation of the water damage resistance for the micro-surfacing mixture was carried out through assessing the wet wheel wear value subsequent to a 6-day immersion period. The outcomes of this testing are documented in Table 9.

It can be seen from Table 9 that with the increase in TCN composite photocatalytic material content, the 6 d wet wheel wear value showed an upward trend, but it was still less than the 800 g/m^2^, as required by the specification. The 6 d wet wheel wear values when the TCN content was 1%, 3%, 5%, and 7% were increased by 0.8%, 2.4%, 4.3%, and 8.1%, respectively, compared with those without TCN. The observation reveals that the growth rate stayed beneath 10%, suggesting a minimal impact of TCN addition on the micro-surfacing mixture’s resistance to water damage. This observed minimal effect is attributed to the dispersion mode of the prepared TCN composite photocatalyst within the emulsified asphalt; it functioned as a modifier, thereby enhancing the asphalt’s properties without significantly affecting its water resilience. Compared with the method of replacing mineral powder in the micro-surfacing mixture, it could be more evenly dispersed and reduce the agglomeration phenomenon. It had good water damage resistance.

#### 4.4.3. Anti-Rutting Deformation Performance Analysis

The evaluation of the micro-surfacing mixture’s resistance to rutting was carried out through the implementation of a rut deformation test. The resultant data from this assessment are presented in Table 10 for further examination.

As evident in Table 10, a decline in both the width deformation rate and rut depth percentage was observable when the concentration of TCN composite photocatalytic material incorporated was below 5%. Compared with the width deformation rate without TCN, the maximum decrease was 8.5%, and the rut deformation rate was up to 4.9%. When the content was more than 5%, there was a gentle or rising trend, which still met the requirements of the specification. It was demonstrated that the incorporation of photocatalytic materials did not have a detrimental influence on the rutting resistance of the micro-surfacing mixture. On the contrary, it had a weak lifting effect. The reason may be that the photocatalytic material was suspended in the asphalt and could form a supporting structure with the asphalt in the micro-surfacing mixture. When the wheel was rolled, it could bear the wheel to a certain extent. The rolling pressure of the micro-surfacing mixture allowed the mixture to exhibit good anti-rutting ability.

#### 4.4.4. Anti-Skid Performance Analysis

The skid resistance characteristics of environmentally friendly micro-surfacing systems incorporating varying concentrations of TCN composite photocatalytic materials were meticulously examined through dual experimental approaches: assessments of pavement structural depth and implementation of the pendulum friction coefficient measurement technique. The findings pertaining to the structural depths and corresponding pendulum values of micro-surfacing mixtures with differing TCN photocatalyst compositions are summarized in Table 11.

From Table 11, it can be seen that the structural depth of the micro-surfacing mixture with five different TCN contents met the requirements of the specification not less than 0.55 mm. With the increase in TCN content, the paving diameter became larger, and the corresponding structural depth decreased. When the TCN content was 1%, 3%, 5%, and 7%, the structural depth was reduced by 2.5%, 3.8%, 5.1%, and 7.6%, respectively, compared with the undoped TCN. It can be seen that the decrease did not exceed 10%, indicating that the addition of TCN had little influence on the structural depth. The pendulum value BPN of the five groups of tests decreased. The pendulum value of the TCN content of 1%, 3%, 5%, and 7% was reduced by 1.1%, 1.3%, 2.5%, and 3.3%, respectively, compared with that without TCN, and the difference was very small, and all met the requirements of the specification. From the analysis of the above two test results, it can be concluded that the addition of TCN photocatalytic material had no influence on the anti-skid property of micro-surfacing.

### 4.5. Effect Analysis of Environmentally Friendly Micro-Surfacing Degradation of Automobile Exhaust

**Figure 16:**
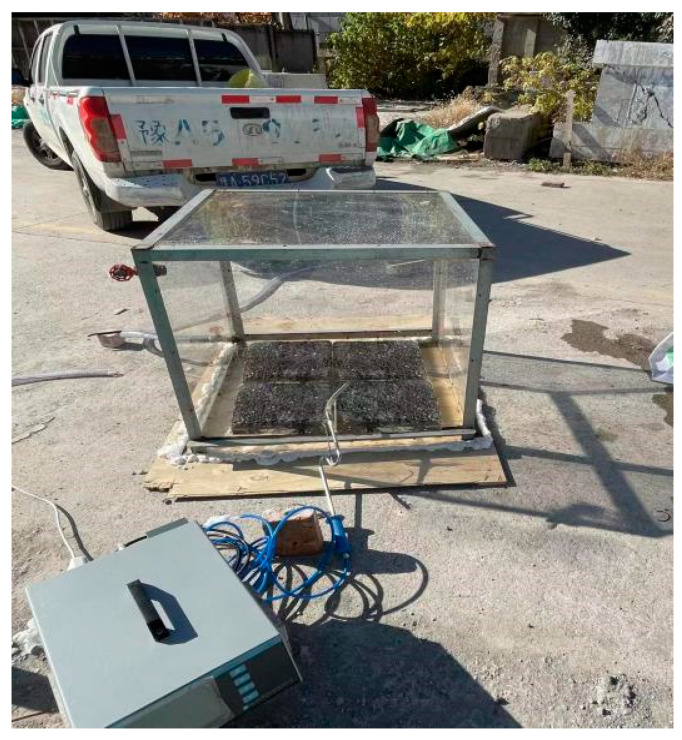
Degradation tail gas test process.

**Figure 17:**
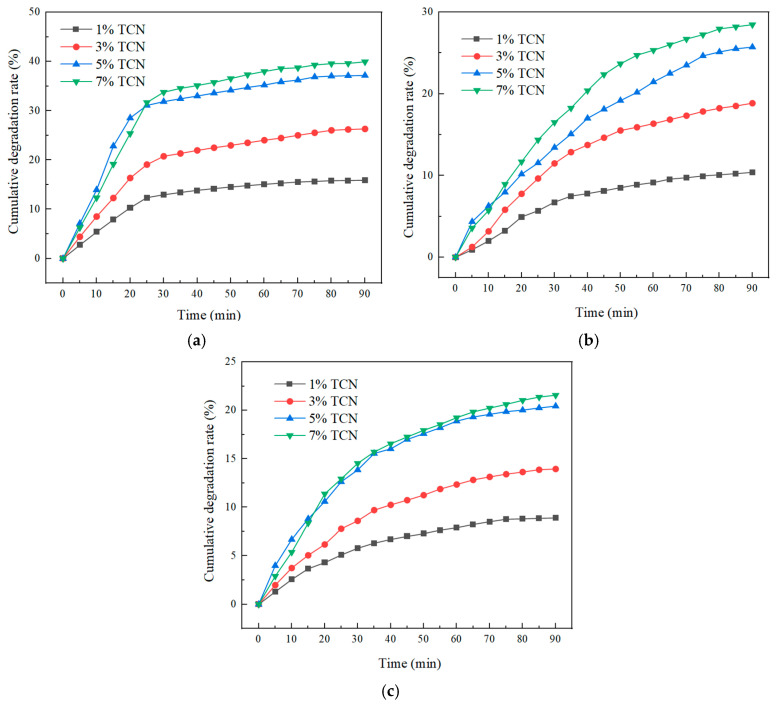
The cumulative degradation rate of automobile exhaust with different TCN contents. (**a**) Cumulative degradation rate of NO. (**b**) Cumulative degradation rate of CO. (**c**) Cumulative degradation rate of HC.

Figure 16 illustrates the experimental procedure employed to assess the degradation of automobile exhaust within the context of eco-friendly micro-surfacing. With a total testing duration set at 90 min, the data collection commenced at 5 min intervals following the initiation of the experiment. Utilizing Equation (1), as detailed in Section 3.1.2, the gathered data were subsequently analyzed, and the resultant findings are depicted in Figure 17.

Figure 17 indicates that the eco-friendly micro-surfacing asphalt mixture demonstrated a substantial degradation efficacy toward NO, CO, and HC. A noteworthy disparity in degradation rates among these three pollutants emerged when examining the cumulative degradation rates at 90 min. Specifically, NO exhibited a significantly superior degradation rate, reaching 37.16%, compared to CO and HC. Moreover, the figure illustrates an ascending trend in the degradation efficiency of all three gases as the quantity of TCN composite photocatalytic material increased, highlighting the considerable impact of the photocatalyst dosage on the overall degradation performance. However, when the content reached 7%, the cumulative degradation rate of the three gases did not increase significantly compared with the 5% content, which indicates that the micro-surfacing mixture with an appropriate amount of photocatalytic material had a good degradation influence on automobile exhaust gas. When the limit was exceeded, it could not have an infinite improvement effect on the degradation of automobile exhaust, and it also caused the waste of materials. In different reaction time periods, the degradation efficiency of NO by the environmentally friendly micro-surfacing mixture was different. Within the test time of 0 min to 25 min, the degradation effect reached about 80% of the degradation efficiency of 90 min, and raised slowly after 25 min, and finally tended to be gentle. The degradation rates of CO and HC by the environmentally friendly micro-surfacing mixture gradually increased with the increase in TCN dosage. When the dosage was 5%, the degradation rates of CO and HC were 25.72% and 20.44%, respectively, which were 2.47 times and 2.30 times that of the 1% content, and the degradation effect was significantly improved.

### 4.6. Influencing Factors of the Photocatalytic Performance Attenuation of Environmentally Friendly Micro-Surfacing

#### 4.6.1. Influence of the Number of Continuous Tests on Photocatalytic Performance

**Figure 18:**
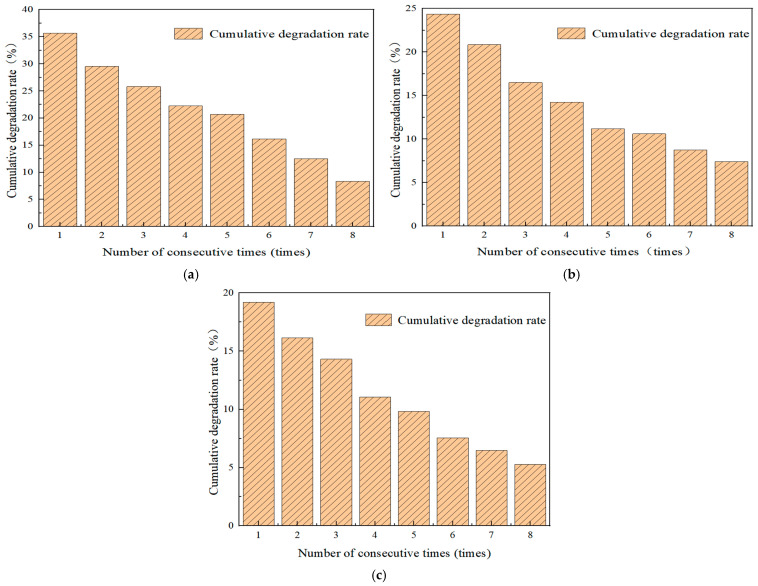
The cumulative degradation efficiency of NO, CO, and HC by continuous test times was studied. (**a**) Cumulative degradation rate of NO. (**b**) Cumulative degradation rate of CO. (**c**) Cumulative degradation rate of HC.

When the TCN composite photocatalytic material decomposes nitrogen oxides, inorganic substances such as nitrates will be generated. When these substances cover the road surface, they will affect the light absorption degree and have a negative impact on the photocatalytic performance. Still, following the test method in Section 2.2, the photocatalytic degradation test of automobile exhaust on the test specimens was carried out. The number of continuous tests was designed to be eight times, and the test duration was 90 min. The evaluation was made through the cumulative degradation efficiency index. The test results are shown in Figure 18.

From Figure 18, it can be concluded that as the number of tests increased, the 90 min cumulative degradation efficiencies of the three gases, NO, CO, and HC, gradually decreased. At the end of the eight time tests, the cumulative degradation rates of NO, CO, and HC decreased from 35.68%, 24.37%, and 19.20% to 8.34%, 7.40%, and 5.29%, respectively. The decrease was relatively large compared to a single time test. This is because the nitrates produced during the degradation of nitrogen oxides by the photocatalytic material covered the surface of the micro-surfacing, reducing the contact area between the photocatalytic material and sunlight, and thus lowering the cumulative degradation rate.

#### 4.6.2. Influence of the Number of Water Flushes on Photocatalytic Performance

**Figure 19:**
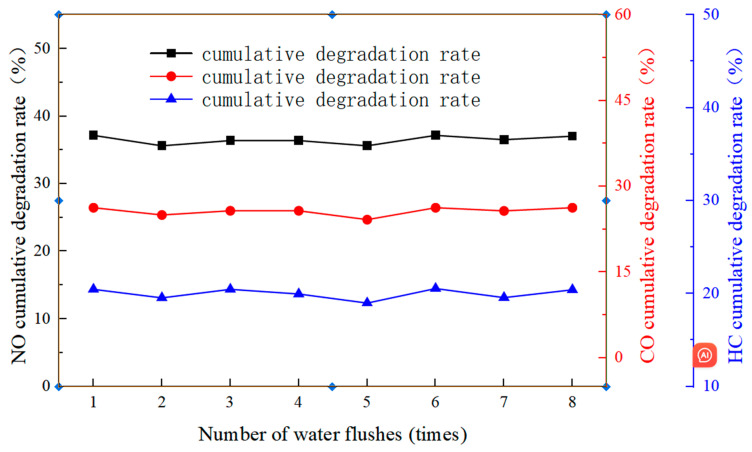
The cumulative degradation rate of NO, CO, and HC by water washing times.

From the continuous tests, it was found that the higher the number of tests, the lower the cumulative degradation rate of the exhaust gas. This is because the nitrates produced during the photocatalytic reaction are easily soluble in water. Therefore, there is no need for scrubbing; simply sprinkling water can dissolve them, and they will volatilize on their own. Based on this, a water flush test was designed in this subsection to observe the influence law on the photocatalytic performance. After a specimen underwent one time photocatalytic degradation, it was simply flushed with water and then dried at room temperature. Then the exhaust gas degradation test was repeated. The test ended after eight repetitions. The test results are shown in Figure 19.

From Figure 19, it can be seen that for the environmentally friendly micro-surfacing mixture, the cumulative degradation rates of the three gases fluctuated slightly under different numbers of water flushes. For example, from the first to the last water flush, the maximum differences in the cumulative degradation rates of NO, CO, and HC were 1.55%, 2.08%, and 1.47%, respectively. It can be concluded that with the increase in the number of flushes, the cumulative degradation rate did not show a gradual attenuation trend. It can be considered that the photocatalytic activity of the environmentally friendly micro-surfacing designed in this paper will not decrease with the increase in the number of water flushes, and its photocatalytic performance can be restored to the original level after being washed by water.

## 5. Conclusions

(1) The TiO_2_/g-C_3_N_4_ (TCN) composite photocatalyst was prepared using the high-temperature calcination method with commercial anatase TiO_2_ and melamine as raw materials. The optimum doping ratio of TCN was obtained by using the cumulative degradation rate of NO, CO, and HC as an evaluation index, and its microscopic characterization was carried out. When the mass ratio of TiO_2_ and melamine was 1:4, the calcination temperature was 550 °C, and the calcination time was 4 h, and the prepared TCN composite photocatalyst had strong photocatalytic activity.

(2) The particle size of TiO_2_ nanoparticles was very small, the surface energy was large, and it was easy to agglomerate. The g-C_3_N_4_ was lamellar, and the surface was relatively smooth. The TiO_2_ particles in the TCN composite were evenly distributed on the surface of the g-C_3_N_4_ sheet structure, and the small particles showed a highly dispersed state, indicating that g-C_3_N_4_ inhibited the agglomeration of TiO_2_. The crystal structure of TiO_2_ and g-C_3_N_4_ was not damaged during the synthesis process. The g-C_3_N_4_ inhibited the agglomeration of TiO_2_. The introduced N-Ti bond changed the electronic structure of TiO_2_, narrowed the band gap and broadened the visible light response range.

(3) After adding TCN, a dose-dependent rise in the softening point of SBR-modified emulsified asphalt was observed within the 0% to 7% concentration increment, accompanied by a decrease in penetration. Conversely, the aged composite’s penetration ratio and ductility ratio escalated alongside an increment in TCN dosage within the same range, while the softening point increment diminished. Notably, at a 9% dosage, a reversal of this trend emerged, suggesting an optimal TCN content that favorably enhances both the high-temperature performance and anti-aging characteristics of SBR-modified asphalt. However, a gradual decline in the ductility of the composite emulsion with increasing TCN levels alludes to a detrimental impact on the low-temperature performance of the SBR-modified emulsion. Of particular concern, when the TCN concentration hit 9%, the storage stability of the composite over 1-day and 5-day periods fell short of the specified standards. Consequently, through comprehensive evaluation, the suitable TCN content range was narrowed down to 1% from 7%.

(4) The optimum water consumption of micro-surfacing mixture with and without photocatalytic materials was 7% and 6%, respectively, the optimum cement content was 2%, and the optimum oil–stone ratio was 7.2%. The addition of TCN improved the wear resistance and rutting resistance of the micro-surfacing mixture, and had little effect on the water damage resistance and skid resistance, but still met the requirements of the specification.

(5) The environmentally friendly micro-surfacing pitch mixture had a sensible degradation result on NO, CO, and HC. From the cumulative degradation rate at 90 min, there was a big difference in the degradation rate of the three gases. The degradation rate of NO was significantly higher than that of CO and HC. With the increase in the content of the TCN composite photocatalytic material, the degradation efficiency of the three gases is on the rise. The degradation rates of NO, CO, and HC by 5% dosage were 37.16%, 25.72%, and 20.44%, respectively, which were 2.34 times, 2.47 times, and 2.30 times that of the 1% dosage, and the degradation effect was obviously improved.

## Figures and Tables

**Table 1:** Technical index of TiO_2_.

Target of Test	Average One Grain per Time Diameter (nm)	Specific Surface Area (m^2^/g)	Bulk Density (g/cm^3^)	Purity (%)	Water Content (%)
Test result	13	82	0.36	98.3	1.8

**Table 2:** SBR-modified emulsified asphalt technical index requirements.

Test Indicator		Unit	Test Result	Technical Standard
The remaining amount on the sieve		%	0.03	≤0.1
Enguera viscosity		-	12.8	3–30
Properties of evaporation residue	Evaporation residue content	%	61.6	≥60
	Needle penetration	0.1 mm	64.4	40~100
	Softening point	°C	54.3	≥53
	Ductility (5 °C)	cm	49.1	≥20
	Solubility	%	99.5	≥97.5
Storage stability	1 d	%	0.63	≤1
	5 d	%	2.61	≤5

**Table 3:** Technical indexes of coarse and fine aggregate for micro-surfacing.

Aggregate	Test Indicator (Units)	Standard Requirement	Test Result		Test Method
			Basalt	Limestone	
Coarse aggregate	Stone crushing value (%)	≤26	12.7	—	T0316
	Los Angeles weared value (%)	≤28	15.3	—	T0317
	Polished drum coating stone value BPN	≥42	56.2	—	T0321
	Ruggedness (%)	≤12	9.5	—	T0314
	Needle-like content (%)	≤15	10.4	—	T0312
Fine aggregate	Ruggedness (%)	≤12	—	9.5	T0340
Mineral material	Sand equivalent (%)	≥65	77.1	77.8	T0334

**Table 4:** Technical index of cement.

Test Indicator		Unit	Test Result	Technical Requirement
Specific surface area		m^2^/kg	365	≥300
Stability		—	qualification	qualification
Setting time	Initial setting time	min	159	≥45
	Final setting time		276	≤600
Compressive strength	3 d	MPa	30.7	≥17.0
	28 d		48.3	≥42.5
Break off strength	3 d	MPa	5.1	≥3.5
	28 d		8.6	≥6.5

**Table 5:** Initial concentration values of each gas.

Gas	HC (ppm)	CO (%)	NO (ppm)
Initial concentration control	130~160	0.90~1.20	80~110

**Table 6:** The effect of TCN content on the performance of modified emulsified asphalt.

Test Indicator		Unit	TCN Latex Content				
			1%	3%	5%	7%	9%
Evaporated residue	Needle penetration	0.1 mm	64.1	62.9	60.5	58.7	57.9
	Softening point	°C	55.4	55.9	57.1	58.2	58.5
	Ductility(5 °C)	cm	47.2	42.4	33.8	30.6	28.1
Storage stability	1 d	%	0.65	0.72	0.81	0.93	1.37
	5 d	%	2.88	3.49	3.85	4.26	5.12

**Table 7:** Composite-modified emulsified asphalt evaporation residue aging test results.

TCN Composite Photocatalytic Material Content (%)	25 °C Penetration (0.1 mm)	Softening Point (°C)	5 °C Ductility (cm)
0	44.5	61.2	33.4
1	45.4	61.6	32.5
3	45.8	60.5	30.2
5	44.2	61.3	24.8
7	43.9	61.7	22.6
9	42.6	62.2	20.4

**Table 8:** The 1 h wet wheel wear value of the mixture with different TCN material contents.

TCN Composite Photocatalytic Material Content (%)	0	1	3	5	7	Specification Requirement
1 h WTAT (g/m^2^)	458.2	446.7	440.5	431.8	469.4	≤540

**Table 9:** The 6 d wet wheel wear value of the mixture with different TCN material contents.

TCN Composite Photocatalytic Material Content (%)	0	1	3	5	7	Specification Requirement
6 d WTAT(g/m^2^)	624.6	630.1	639.4	651.7	675.2	≤800

**Table 10:** The deformation rate of the mixture with different TCN material contents.

TCN Composite Photocatalytic Material Content (%)	0	1	3	5	7	Specification Requirement
PLD (%)	4.7	4.6	4.5	4.3	4.3	≤5.0
PVD (%)	10.2	10.0	9.9	9.7	9.8	-

**Table 11:** The structural depth and pendulum value of the mixture with different TCN material contents.

TCN Composite Photocatalytic Material Content (%)	0	1	3	5	7	Specification Requirement
Texture depth (mm)	0.79	0.78	0.76	0.75	0.73	≥0.6
Pendulum value	51.9	51.4	51.2	50.6	50.2	≥45

## Data Availability

Some or all data, models, or code that support the findings of this study are available from the corresponding author upon reasonable request.
